# Lignin nanoparticles as co-stabilizers and modifiers of nanocellulose-based Pickering emulsions and foams

**DOI:** 10.1007/s10570-023-05399-y

**Published:** 2023-07-29

**Authors:** Melissa B. Agustin, Neda Nematollahi, Mamata Bhattarai, Erfan Oliaei, Mari Lehtonen, Orlando J. Rojas, Kirsi S. Mikkonen

**Affiliations:** 1 Department of Food and Nutrition, Faculty of Agriculture and Forestry, University of Helsinki, P.O. Box 66, FI-00014 Helsinki, Finland; 2 Department of Bioproducts and Biosystems, Aalto University, P.O. Box 16300, 00076 Aalto, Finland; 3 Wallenberg Wood Science Center, Department of Fiber and Polymer Technology, KTH Royal Institute of Technology, SE-100 44 Stockholm, Sweden; 4 Bioproducts Institute, Department of Chemical and Biological Engineering, Department of Chemistry and Department of Wood Science, University of British Columbia, 2360, East Mall, Vancouver, BC V6T 1Z3 Canada; 5 Helsinki Institute of Sustainability Science, University of Helsinki, P.O. Box 65, FI-00014 Helsinki, Finland; 6 Present Address: VTT Technical Research Centre of Finland Ltd., P.O. Box 1000, FI-02044 Espoo, Finland

**Keywords:** Pickering emulsions, Nanocellulose, Lignin nanoparticles, Foams, Adsorption, Pharmaceutical pollutants

## Abstract

Nanocellulose is very hydrophilic, preventing interactions with the oil phase in Pickering emulsions. This limitation is herein addressed by incorporating lignin nanoparticles (LNPs) as co-stabilizers of nanocellulose-based Pickering emulsions. LNP addition decreases the oil droplet size and slows creaming at pH 5 and 8 and with increasing LNP content. Emulsification at pH 3 and LNP cationization lead to droplet flocculation and rapid creaming. LNP application for emulsification, prior or simultaneously with nanocellulose, favors stability given the improved interactions with the oil phase. The Pickering emulsions can be freeze–dried, enabling the recovery of a solid macroporous foam that can act as adsorbent for pharmaceutical pollutants. Overall, the properties of nanocellulose-based Pickering emulsions and foams can be tailored by LNP addition. This strategy offers a unique, green approach to stabilize biphasic systems using bio-based nanomaterials without tedious and costly modification procedures.

**Supplementary Information:**

The online version contains supplementary material available at 10.1007/s10570-023-05399-y.

## Introduction

Pickering emulsions (PEs) are emulsions that use solid particles as stabilizers of the oil–water interface. Although the phenomenon was described earlier by Ramsden in 1903, (Ramsden 1903) the emulsion was named after S.U. Pickering, who demonstrated the emulsification of paraffin oil in water aided by basic insoluble sulfates and other types of solids in 1907 (Pickering 1907). About a century after their work, there has been a renewed and continuing interest in PE, reflected in the increasing number of publications since the beginning of the twenty-first century (Scopus database, Fig. S1). The interest may have stemmed from the exceptional high stability of PEs and the recent advances in particle synthesis, which lead to unique applications, not possible with conventional surfactant-based emulsions.

PEs are highly stable systems due to the nearly irreversible adsorption of the solid particles at the droplet interface (Arditty et al. 2003; Binks 2002). Unlike conventional emulsions, where the surfactants form a thin molecular film around the droplets, a thick particle layer surrounds the droplets in PEs, acting as a physical barrier against destabilization (Binks 2002; Tambe and Sharma 1993). Owing to high droplet stability and compared with ordinary emulsions, PEs can efficiently encapsulate and protect active ingredients for an extended time (Cui et al. 2021; Haji et al. 2022). The stable PE droplets can also act as micro-reactors for catalytic processes by providing interfacial reaction sites, much more than those achieved in classical biphasic systems (Chang et al. 2021; Ni et al. 2022; Zhang et al. 2022a, b). Having the possibility to maintain the droplet structure even after the removal of the liquid phases makes PEs effective templates for the synthesis of hollow capsules, and porous beads or monoliths (Capron and Cathala 2013; Mudassir, et al. 2021; Pascaud et al. 2022; Rodriguez and Binks 2019). Evidently, the novel applications of PEs are feasible because of the wide variety of particle stabilizers made available recently. The breakthroughs in particle synthesis have enabled the production of particles of varying surface properties, dimensions, and shapes (spheres, rods, discs, fibrils, cubes, etc.) (Baig et al. 2021). Particles that physically or chemically transform under different conditions give rise to stimuli-responsive PEs for controlled-delivery systems, catalysis, and oil recovery, among many other applications (Rodriguez and Binks 2020; Tang et al. 2015).

The remarkable progress in particle synthesis allows diverse sources of Pickering stabilizers. Some compounds that are bound to complex matrices are now possible to be extracted and transformed into micro- or nanoparticles of uniform size and shape using top–down approaches. The opposite also applies to small soluble compounds via bottom-up synthesis. Combining these techniques in the quest to produce Pickering stabilizers that are non-toxic, biocompatible, and biodegradable, especially for food and biomedical applications, has led to the utilization of bio-based raw materials. Moreover, with the global call to reduce carbon emissions, tapping renewable and sustainable materials in the production of bio-based Pickering stabilizers has drawn significant attention (Calabrese et al. 2018; Hossain et al. 2021; Zhang et al. 2022a, b).

Nanocellulose and lignin nanoparticles (LNPs) are bio-based nanomaterials often derived from wood and other plant biomass. Nanocelluloses are often produced via a top-down approach through combinations of mechanical, chemical, and enzymatic treatments of cellulosic pulp. They vary from the highly crystalline rod-like cellulose nanocrystals (CNCs) to the highly entangled, high-aspect ratio cellulose nanofibers (CNF) (Heise et al. 2021). There is also microbial cellulose, which is composed of nanostructured twisted ribbons of pure cellulose produced by specific bacteria (Hirai et al. 1998). LNPs are colloidal particles derived from lignin, one of the major side stream materials of pulp and paper industries. LNPs are produced in various shapes and sizes, and they have considerably different morphology and surface properties when compared to raw lignin (Beisl, et al. 2017; Iravani and Varma 2020; Österberg et al. 2020). Among the various methods of preparing LNPs, anti-solvent nanoprecipitation, producing spherical LNPs, is the most commonly used (Figueiredo et al. 2021; Österberg et al. 2020).

Both nanocelluloses and LNPs proved to be effective in stabilizing PEs (Bai et al. 2019; Capron et al. 2017; Zhu et al. 2021). While nanocellulose is generally considered highly hydrophilic, it exhibits a certain degree of amphiphilicity because of the varying distribution of hydroxyl and methine groups on the different crystal planes of nanocellulose (Lehtio et al. 2002; Mazeau 2011; Moreau et al. 2016). This unequal exposition of surface chemical groups enables nanocellulose to adsorb at oil/water interfaces. However, it has to be noted that this mainly applies to cellulose nanocrystals while the situation for nanofibrillar cellulose is more complex, considering the surface chemical composition (hardly pure cellulose) and the presence of disordered cellulose domains. For related reasons, fibrillated cellulose is not a good emulsifier but a good emulsion stabilizer. Surface charge and aspect ratio greatly influence the efficiency of nanocelluloses as emulsion stabilizers (Capron et al. 2017; Fujisawa et al. 2017; Parajuli and Urena-Benavides 2022). However, enhancing further their wettability in the oil phase requires chemical modifications that introduce hydrophobic functionalities. Some hydrophobization techniques for nanocelluloses include acylation or esterification (Dudefoi et al. 2022; Sebe et al. 2013; Tang et al. 2019), carboxylation followed by amidation with long-chain alkylamines (Zhang et al. 2017), or reaction with long-chain quaternary ammonium salts (Saidane et al. 2016), and hydrophobic polymer grafting (Zoppe et al. 2012). Lignin, on the other hand, is inherently amphiphilic because of the presence of hydrophobic and hydrophilic groups in its structure (Österberg et al. 2020). However, their heterogeneity, both in chemical structure and morphology, has limited their potential as PE stabilizers. Producing them as LNPs that can uniformly be dispersed in water enhanced their performance as PE stabilizers, which was affected by their source, dimension, and concentration (Ago et al. 2016; Sipponen et al. 2017).

Previous work on nanocelluloses and LNPs as PE stabilizers examined their performance mostly as a single stabilizer. Recently, the concept of dual stabilizers, especially the combination of nanocelluloses with other particles, has shown some advantages. A synergistic stabilizing effect was observed when cellulose nanocrystals were combined with pea protein isolate or zein colloidal particles (Nie et al. 2022; Wei et al. 2021). Hetero-aggregation effects and depletion stabilization were observed in the combinations of cellulose nanofibrils with nanochitin and cellulose nanocrystals, respectively (Bai et al. 2018; Guo et al. 2023). Additional functionalities to the foams templated from PEs, such as photocatalytic activity or conductivity, were also achieved when nanocellulose is coupled with titanium oxide or carbon nanotubes (Mougel et al. 2019; Voisin et al. 2021). The combination of nanocellulose and LNPs as dual stabilizers, to our knowledge, has not been explored, although there were few studies (Ferreira et al. 2022; Guo et al. 2021) on the effect of residual lignin on nanocellulose in stabilizing PEs. Thus, in this study, we combined LNPs and nanocellulose as stabilizers in the preparation of oil-in-water PEs. Our hypothesis is that the addition of LNPs will affect the hydrophilic/hydrophobic balance at the interface (Suzuki et al. 2022), thereby, producing more stable PEs than those stabilized by nanocellulose alone. With the combined stabilizers, hydrophobization could be achieved without additional chemical modification, and with the ability to control the amount of LNPs, the surface energy balance can be tuned, and so were the properties of the emulsion. Moreover, the addition of LNPs is envisioned to provide functionalities to the PE-templated foams, when used as potential adsorbents in water treatment applications. To test our hypotheses, we prepared PEs stabilized by TEMPO-oxidized cellulose nanofibers (TCNF) and spherical colloidal LNPs and investigated the effect of various factors, including the LNP concentration and charge, pH of the aqueous phase, and order of mixing the stabilizers on the properties of the emulsions. TCNF was chosen because our prior investigation using different nanocelluloses showed that TCNF had the best performance to adsorb pharmaceuticals, which are the target pollutants when the subsequent PE-templated foams are used as adsorbents (Agustin et al. 2022). The interest in pharmaceutical pollutants is motivated by the surging global concern arising from the well-documented deleterious effects of pharmaceutical pollution on the health of humans and ecosystems (Ortuzar et al. 2022). Thus, this study contributes to a fundamental understanding of the stabilization of PEs with TCNF and LNPs, and offers a potential means of addressing pharmaceutical pollution by tapping renewable wood-based nanomaterials.

## Experimental section

### Materials

Never-dried softwood kraft pulp fibers (Metsä Board Husum mill, Sweden) were used to prepare TCNF following the method of (Saito et al. 2007). Sulfur-free birch lignin produced by BLN process (named after the company that initially developed the technology) was provided by CH Bioforce Oy (Finland). Briefly, the process involves hot water (150 °C) extraction of hemicelluloses from biomass followed by alkali cooking to extract lignin, all in oxygen-starved conditions (von Schoultz 2015). The LNPs were prepared by anti-solvent nanoprecipitation in acetone and water (Figueiredo et al. 2021). These LNPS were cationized by coating with glycidyltrimethylammonium chloride (GTAC)-treated lignin to produce cationic LNPs (cLNPs) (Agustin et al. 2022; Sipponen et al. 2017). The detailed preparation of TCNF, LNPs, and cLNPs and their basic characteristics are available in the Supporting Information (Fig. S2). Hexadecane, GTAC, cyclohexanone, and acetone were purchased from Fisher Scientific (Finland). The pharmaceutical compounds, which are certified reference materials, dialysis membrane tubing (Spectra/Por 7, 6–8 kDa molecular weight cut-off (MWCO), Spectra/Por 1, 1 kDa MWCO), glass microfibre filters with a 0.7 µm pore size (Whatman GF/F), chromasolv-grade acetonitrile and methanol were obtained from Sigma-Aldrich (Finland).

### Emulsion preparation

Different formulations of oil-in-water PEs were prepared using different combinations of TCNF with LNPs or its cationic form, cLNPs, as shown in Table 1. Emulsions with only LNPs or TCNF used as stabilizers were also prepared as controls. For clarity, all percentages of the components indicated from this point forward refer to weight percentages relative to the total weight of the emulsion, unless otherwise stated. The emulsions were named with T, L, and cL corresponding to TCNF, LNPs and cLNPs, respectively. The numbers that follow the letters were the weight percentages of each respective nanomaterial in terms of their dry weight and were calculated from the solid content of the suspension. For emulsions with combined stabilizers, the percentage of TCNF, which was constant at 0.1% was not indicated for simplicity. All formulations had 10% hexadecane as the oil phase. From the different combinations, the most stable emulsion was chosen for studying the effect of pH and the order of mixing the stabilizers.

**Table 1:** The formulation of the different Pickering emulsions prepared

Stabilizers	Emulsion code	TCNF (%)*	LNP (cLNP) (%)	Oil (%)
TCNF-LNP	TL.05	0.10	0.05	10
	TL.10	0.10	0.10	10
	TL.20	0.10	0.20	10
TCNF-cLNP	TcL.05	0.10	0.05	10
	TcL.10	0.10	0.10	10
	TcL.20	0.10	0.20	10
only LNP	L.10	0	0.10	10
only TCNF	T.10	0.10	0	10

*All percentages refer to weight percentage with respect to the total weight of the emulsion

Before each emulsion preparation, the TCNF and LNP suspensions were vigorously stirred or shaken to disperse sedimented particles or break any flocs that formed during storage. The calculated weight of the suspension corresponding to the target dry weight percentage of the nanomaterials was combined with milliQ water to produce the aqueous phase, which corresponds to 90% of the total weight of the emulsion. This aqueous phase was kept in a capped bottle and magnetically stirred overnight to completely disperse the nanomaterials. The pH was then adjusted to the desired pH (3, 5, or 8) by adding dropwise 0.1 M HCl or 0.1 M NaOH while the pH was monitored by a meter (Mettler Toledo S220, Fisher Scientific, Finland). The corresponding 10% weight of hexadecane was then added and the mixture was pre-emulsified using the Ultra-Turrax (T-18 basic, IKA, Staufen, Germany) equipped with a 25-mm disperser-type stirrer at 16,000 rpm for 5 min. The coarse emulsion was further sheared in a Microfluidizer 110Y (Microfluidics, MA, USA) with four passes at 800–850 bar to obtain the final emulsion. For the order of mixing, the emulsion containing only one type of stabilizer was first prepared in the same way as described taking into consideration the concentration of each component in the final emulsion. Then, the second stabilizer was added to the existing emulsion, and the mixture was further mixed using the Ultra-Turrax at 8000 rpm for 3 min.

### Emulsion characterization

The droplet morphology was visualized using the optical light microscope (Axiocam MR3 camera) (Carl Zeiss Inc., Oberkochen, Germany). A diluted emulsion was dropped on a clean glass slide and observed at 40 or 100 × optical magnification.

The droplet size distribution and the volume-average droplet size, D_*(4,3)*_ were measured by laser diffraction using the Mastersizer Hydro 3000 SM with a Hydro EV suspension accessory (Malvern Instruments Ltd, Worcestershire, UK). Refractive indices of 1.43 and 1.33 were used for hexadecane and water, respectively. Each sample measurement was done immediately after preparation and during storage for 30 days.

The zeta potential (ζ) of the emulsions at 25 ℃ was determined by dynamic light scattering using the Zetasizer Nano ZS (Malvern Instruments, Worcestershire, UK). A 50-µL emulsion was diluted with deionized water to obtain a turbid suspension and the measurements were performed by loading the diluted samples in folded capillary cells. Smoluchowski equation was used to calculate the ζ from the electrophoretic mobility data using Suspension Technology Software v. 5.10 (Malvern Instruments, Worcestershire, UK).

The emulsion viscosities were measured using a HAAKE MARS 40 rheometer (Thermo Scientific, USA) at 20 ℃ with a 35-mm diameter parallel plate. The viscosity at a shear rate range of 20 to 120 s^−1^ was determined in duplicated samples.

The physical stability of the emulsions was monitored by a Turbiscan Lab expert (Formulaction, Toulouse, France) for 30 days. The transparent glass vials containing 20 mL of emulsions were scanned from the bottom to the top by a near-infrared light source at 880 nm. The average height of the emulsion in the vial was approximately 40 mm. The changes in intensities of transmitted and backscattered lights measured over the entire height of the samples are summed up in the parameter called Turbiscan Stability Index (TSI), derived using the built-in software (Turbiscan software v.1.2). All measurements were done in triplicates.

### Preparation and characterization of the Pickering foams and adsorption capacity determination

Two types of foams, one with only TCNF, i.e. without LNPs (TL0) and one with a dry weight ratio of LNP to TCNF of 0.8:1 (TL.8) were prepared. This ratio was chosen based on our previous findings with cLNP/TCNF-based cryogels that the dry weight ratio of cLNPs to TCNF must be 0.6 or higher to achieve significant improvement in the adsorption potential of pharmaceuticals (Agustin et al. 2023). This ratio is also within the range of LNP:TCNF ratios (0.5–2) used to prepare the PEs in the first part of the study. Because of the high water content of the TCNF and LNP suspensions, emulsions with an LNP:TCNF ratio higher than 0.8 was not feasible to prepare, especially when the TCNF content of the emulsion was increased to 0.4% and the oil phase used was 30%. The amount of TCNF is based on a preliminary test of freeze drying TCNF suspensions with different TCNF content. Cyclohexane was used as the oil phase because it can be easily removed by freeze drying. Because of the increase in the solid and oil content, the starting mixture was too viscous to pass through a microfluidizer, thus the emulsification was done only by high-speed mixing using the Ultra-Turrax (T-18 basic, IKA, Staufen, Germany). The mixing speed was increased stepwise from 5000 to 18,000 rpm within a 6-min period and held constant for 2 min more. The emulsion droplet was imaged using an optical microscope, and the rest of the emulsions were frozen in liquid nitrogen and freeze–dried for two days to obtain the foams. The morphology of the foam was visualized using field-emission SEM Sigma VP (Zeiss Instruments, Jena, Germany). A small piece of the foam was sputtered with a 4 nm thick layer of platinum–palladium and imaged in a vacuum at an accelerating voltage of 3 kV. The adsorption potential of the foams towards different pharmaceuticals was determined using the optimized method we described in our previous report (Agustin et al. 2022). Briefly, a multi-component solution with six pharmaceuticals each at 20 mg/L was used as the model system to determine the adsorption capacity of the foams. The adsorption conditions were as follows: 1:1 ratio of the mass (in mg) of the foam to volume (mL) of the pharmaceutical solution, pH 6, 2.5-h contact time, room temperature, and mechanical mixing at 100 motions per minute. After the adsorption, one mL of the solution was withdrawn and filtered (0.45 µm, Acrodisc wwPTFE Membrane), and the residual pharmaceuticals were analyzed by ultra-high performance liquid chromatography (UHPLC) combined with UV/VIS detection. Comparison of the average adsorption potential of TL.8 and TL0 for each pharmaceutical was analyzed by a simple t-test at 0.05 level of significance using Origin Lab Software.

## Results and discussion

### Properties of emulsions stabilized with LNPs and TCNF

Morphology, dimension, wettability, and surface charge of the solid particles influence their capacity to stabilize PE (Gonzalez Ortiz et al. 2020; Low et al. 2020). Here, TCNF and LNPs as stabilizers differ in their morphology and surface properties. The TCNF, with an average width of 6.8 nm and length of a few to several microns, are high-aspect ratio and 1D anisotropic particles (Capron et al. 2017), whereas the spherical LNPs with an average hydrodynamic diameter of 132 nm are 3D isotropic particles. The TCNF has a more negative (higher negative ζ) and more hydrophilic surface than the LNPs. These differences, indeed, affected the initial properties and storage behavior of the PEs stabilized by TCNF and LNPs.

**Fig. 1:**
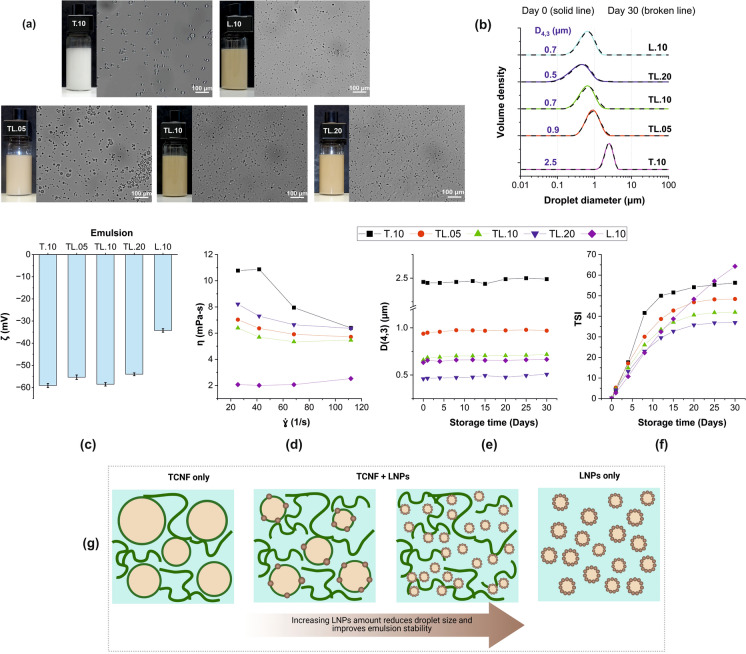
The initial properties and storage behavior of Pickering emulsions stabilized by only TCNF (0.1%, T.10), only LNPs (0.1%, L.10), and their combinations with increasing LNP content (TL.05, TL.10, TL.20): appearance and droplet morphology (**a**), droplet size distribution (**b**), zeta potential, ζ (**c**), dynamic viscosity, η (**d**), change in the volume-average droplet size, D_*(4,3)*_ (**e**) and Turbiscan Stability Index, TSI (**f**), and infographic of the effect of LNPs on TCNF-stabilized emulsions (**g**)

The freshly prepared emulsions stabilized by a single component (LNPs or TCNF) or by dual systems at different combinations appeared homogeneous, without an indication of non-emulsified oil on the surface. Those with LNPs were brown and became darker with the increased LNP content (Fig. 1a). All the emulsions showed individual droplets that were monomodal (Fig. 1a and 1b). The emulsion with only TCNF (T.10) had the narrowest distribution but the highest D_*(4,3)*_ (2.5 µm). At similar concentrations with only LNPs (L.10), the droplets were smaller (D_*(4,3)*_ = 0.7 µm), indicating better performance of LNPs over TCNF as Pickering stabilizers. This could be due to the difference in morphology and surface properties of LNPs and TCNF, as mentioned earlier. The amphiphilic LNPs interact with the highly hydrophobic hexadecane more effectively compared with TCNF. Moreover, the adsorption of the spherical isotropic LNPs at the interface possibly occurs faster than that of the thread-like, highly intertwined anisotropic TCNF. When the LNPs and TCNF were combined, even at the lowest LNP content of 0.05%, the droplet size was significantly reduced to 0.9 µm. With increasing LNP content, the droplet size showed a decreasing trend, with the smallest D_*(4,3)*_ of 0.5 µm observed in TL.20. The decrease in droplet size in the presence of LNPs is attributed to the changes in the hydrophilic/hydrophobic balance the interface. As the amount of LNPs increased, the particle system hydrophilicity was reduced, and the number of particles capable of stabilizing a large interfacial area was increased, producing smaller droplets, as also observed in previous studies (Ago et al. 2016; Kimiaei, et al. 2022). Furthermore, with an increasing number of particles, the kinetics of adsorption of the particles at the oil–water interface increased, resulting in smaller droplet size and higher stability (Ago et al. 2016). These results suggest that depending on the amount of LNPs, the emulsion can be tuned to the desired droplet size.

The ζ of all the emulsions was less than − 30 mV, which is a favorable ζ for stable emulsions (Fig. 1c) (Low et al. 2020). The ζ of the emulsions with combined stabilizers was influenced more by TCNF (− 64 mV) than by LNPs (− 41 mV). Despite having twice the amount of LNPs (0.20%) compared to TCNF (0.10%), the TL.20 system had a ζ of − 54 mV. Meanwhile, the emulsion with only LNPs (L.10) had ζ = − 34 mV.

The dynamic viscosity of the emulsions showed slight shear thinning behavior (Fig. 1d) and the effect of TCNF was evident. T.10 had the highest viscosity attributed to the network of long nanofibrils at the continuous phase. As expected, L.10 had the lowest viscosity as spherical particles have less resistance to flow than fibrillar ones. The emulsions with combined LNPs and TCNF had viscosity values in between those stabilized by only TCNF (T.10) and only LNPs (L.10).

With storage, all the emulsions demonstrated exceptional droplet stability typical of PE and both TCNF and LNPs proved to be effective in protecting the droplets. Neither flocculation nor coalescence occurred during the 30 days of storage (the droplet size distribution and D_*(4,3)*_ remained unchanged, Figs. 1b and 1e). This could be attributed to the highly negative ζ of the droplets and possibly to the efficient adsorption of LNPs and TCNF at the droplet interface. However, changes in the homogeneity of the emulsion with time were observed as reflected in the increasing TSI values (Fig. 1f). The backscattered light decreased at the bottom of the vial (Fig. S3a) while the transmitted light increased (Fig. S3b), indicating creaming driven by density differences between the oil droplets and the water phase. Among the TCNF-containing emulsions, the one with only 0.10% TCNF (T.10), despite being the most viscous, had the highest TSI value. This is attributed to its large droplet size that drives creaming at a faster rate than those with smaller droplets. With increasing LNP content, the TSI followed a decreasing trend parallel to the droplet size, evidently showing TL.20 to be the most stable. The creaming behavior of emulsion with only LNPs (L.10) differed from that containing TCNF. Although L.10 gave the highest TSI at day 30, the curve did not reach a plateau similar to those containing TCNF. This is likely a result of ongoing changes in the system while the other emulsions have completely formed a stable cream at the top layer. This is supported by the backscattering profiles (Fig. S3a) where all the emulsions, except L.10, seemed to have already reached the same thickness of the cream layer. These results indicate that the creaming behavior of the studied emulsions seemed to be influenced by the increased droplet size and that the increase in viscosity in the presence of 0.10% TCNF was not sufficient to suppress creaming.

Overall, the preliminary findings showed that combining LNPs and TCNF in stabilizing PEs resulted in more stable emulsions than that stabilized by only LNPs or TCNF. With combined stabilizers, the droplets were smaller and the size decreased with increasing LNP content (Fig. 1g). With storage, the emulsions were resistant against flocculation or coalescence but were susceptible to creaming, which was slowed down by the addition of LNPs.

### The effect of pH

**Fig. 2:**
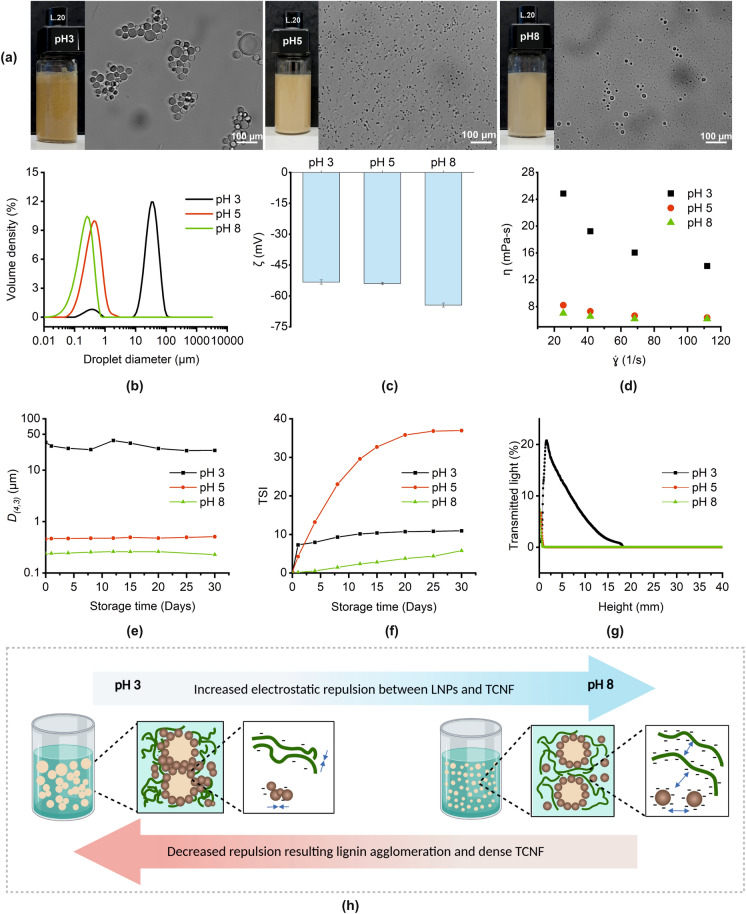
The initial properties and storage behavior of the Pickering emulsions stabilized by 0.1% TCNF and 0.2% LNPs as affected by pH: appearance and droplet morphology (**a**), droplet size distribution (**b**), zeta potential, ζ (**c**), dynamic viscosity, η (**d**), the volume-average droplet size, D_*(4,3)*_ (**e**), and Turbiscan Stability Index, TSI (**f**) and transmission profiles at Day 1 (**g**) and infographic of the effect of pH on LNPs, TCNF and their emulsions (**h**)

Among the PEs with dual stabilizers, TL.20, the sample with the highest LNP content, was selected to explore the effect of pH on stabilization efficiency. The surface properties of the LNPs and TCNF vary with pH because of the ionizable hydroxyls and carboxyls (Agustin et al. 2022), thus it is essential to identify the pH at which their performance as stabilizers is at their full potential. Figure 2 summarizes the different properties of TL.20 at different pH (3, 5, and 8). A pH value higher than 8 was not tested to avoid possible dissolution of LNPs (Agustin et al. 2022; Figueiredo et al. 2021). Creaming was immediately seen at pH 3 and the droplets were flocculated, leading to a bimodal distribution (Figs. 2a, b). This could be due to the agglomeration of LNPs as a consequence of the protonation of the carboxyl groups at pH 3 causing a decrease in ζ (Fig. 2c) as also observed in other studies (Figueiredo et al. 2021; Lievonen et al. 2016). Agglomeration increased the particles size, consequently decreasing the mobility of the LNPs to be adsorbed at the interface (Fig. 2h). There was only a small difference in ζ of the emulsions at pH 3 and 5, and this is possibly because the pH adjustment was done before the emulsification. During microfluidization, it is possible that some carboxyls that were not available on the surface initially became exposed because of the high shearing force. The ζ at pH 8 was the highest because of the deprotonation of the carboxyl groups of the TCNF and LNPs, which further enhanced the repulsive force, making LNPs more available for interfacial adsorption (Fig. 2h). The dynamic viscosity (Fig. 2d) was highest at pH 3 and showed more pronounced thinning behavior than at pH 5 or 8. This can be explained by the behavior of flocculated droplets, which tend to capture some of the continuous phase within their structure, consequently increasing the viscosity (McClements 2015). Prominent thinning with increasing shear stress is also found to be typical for emulsions with flocculated droplets, which are more strongly bound to each other, than those in highly dispersed droplets (Fuhrmann et al. 2022, 2019). The bound droplets act like rigid particles that require high shear stress to disrupt the bonds holding the droplets together, and to make the droplets deform and align with the shear field (Campanella et al. 1995; McClements 2015).

With storage, no significant variation in D_*(4,3)*_ with time was observed at pH 5 and 8, while fluctuations were observed at pH 3 (Fig. 2e). This fluctuation could be due to the random breaking of the flocs to smaller ones when the sample was stirred during the test. Considering the change in TSI values with time, the least stable emulsion seemed to be at pH 5 (Fig. 2f). However, the high TSI values were not supported by the transmission profiles (Fig. 2g), which already show creaming of the pH 3 emulsion from Day 1. The fast creaming rate at pH 3 is attributed to the large flocculated droplets, and when the stable cream was developed, the TSI value remained the same with time. Overall, the pH 3 emulsion was the least stable while the pH 8 emulsion was the most stable, having the highest resistance to creaming because of the small droplets and highly negative ζ. The TSI values of pH 8 emulsion remained low with time and no apparent creaming was visually evident unlike with pH 3. The pH 5 emulsion was not inferior to the pH 8 emulsion and would especially be highly useful in many applications that occur at slightly acidic conditions.

### The effect of the order of mixing the LNPs and TCNF

**Fig. 3:**
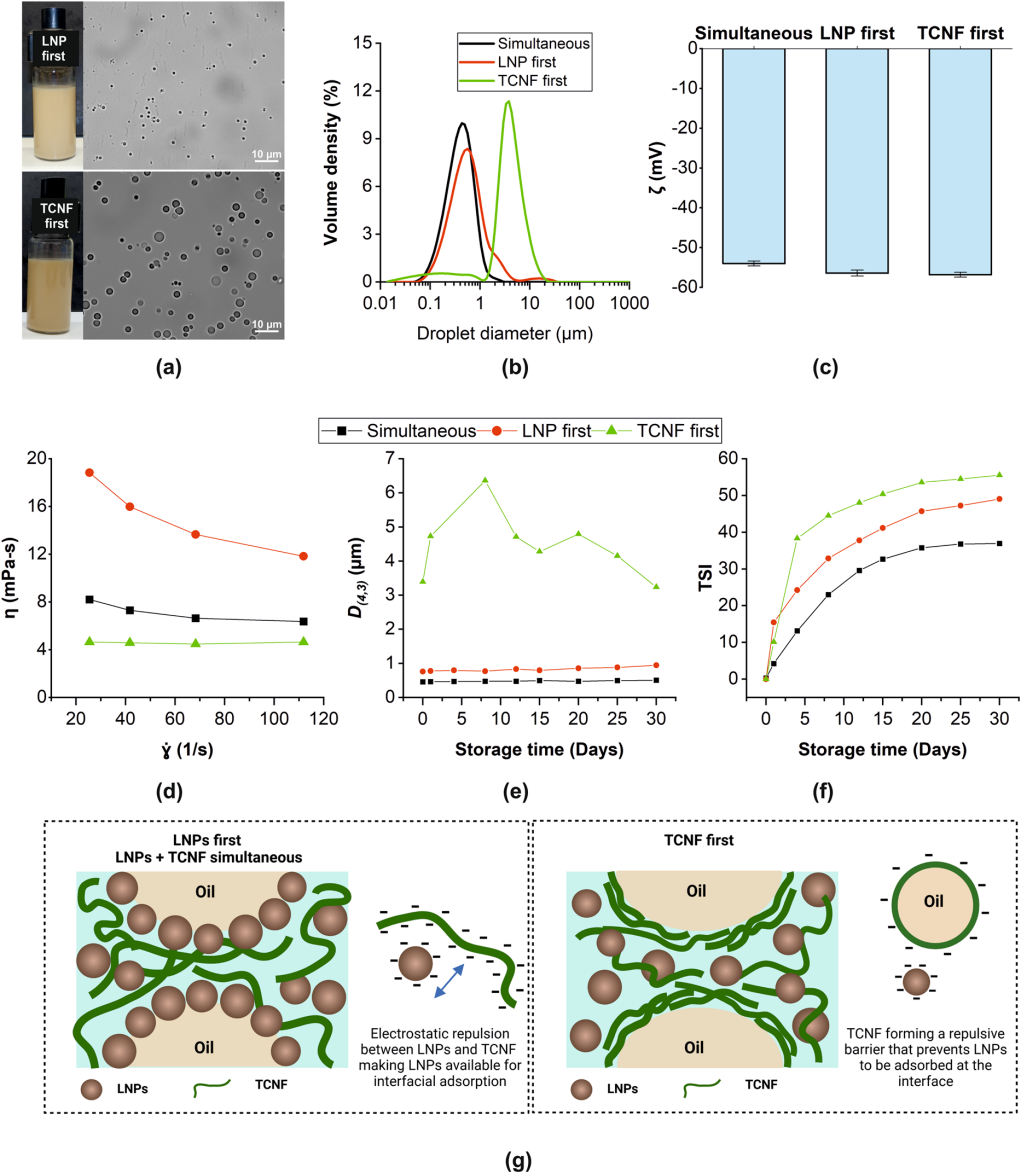
The initial properties and storage behavior of the Pickering emulsions stabilized by 0.1% TCNF and 0.2% LNPs as affected by the order of mixing: appearance and droplet morphology (**a**), droplet size distribution (**b**), zeta potential, ζ (**c**), dynamic viscosity, η (**d**) the volume-average droplet size, D_*(4,3)*_ (**e**) and Turbiscan Stability Index, TSI (**f**), and infographic of the interface (**g**)

The order of mixing the stabilizers in PEs with dual stabilizers influences the assembly of particles at the interface, and the information is useful in tuning the properties of the emulsion (Wei et al. 2021). Here, the order of mixing the LNPs and TCNF was varied and their effect on the emulsion properties was investigated. Using the same formulation as in TL.20, which served as a reference for simultaneous mixing, the LNP-first and TCNF-first emulsions were prepared and named based on the order of mixing. Figure 3 shows the initial properties and storage behavior of the emulsions prepared by sequential mixing in comparison with the simultaneous mixing of stabilizers. Even though the emulsions have the same formulation, there were differences in their initial properties and stability. Sequential mixing resulted in larger droplets (D_*(4,3)*_ = 0.8 and 3.4 µm) than that of simultaneous mixing (D_*(4,3)*_ = 0.5 µm), and the largest droplets with signs of flocculation were observed in TCNF-first emulsion (Fig. 3a, b). The ζ remained highly negative but were not significantly different since the emulsions were prepared at pH 5 and had the same concentration of TCNF and LNPs (Fig. 3c). However, the viscosity (Fig. 3d) varied with the order of mixing, with the highest viscosity observed in the LNP-first emulsion, possibly because the continuous aqueous phase was rich in unadsorbed TCNF. The opposite behavior was observed with TCNF-first, which had the lowest viscosity because the spherical LNPs possibly dominated the continuous phase. Random fluctuations in D_*(4,3)*_ with storage, possibly due to the random breaking of the flocculated droplets, were observed in the TCNF-first emulsion, while an almost constant D_*(4,3)*_ was observed for the other two emulsions (Fig. 3e). As expected, the decrease in viscosity combined with large flocculated droplets promoted creaming for the TCNF-first, making it the least stable, having the highest TSI values (Fig. 3f).

Based on the results, the formation of smaller droplets seemed to be more dependent on the interaction of the amphiphillic LNPs with the hydrophobic hexadecane than that of the interaction with the TCNF. As presented earlier in Section "Properties of emulsions stabilized with LNPs and TCNF", LNPs were more effective in producing smaller droplets than TCNF at similar concentrations and emulsification conditions. Thus, when LNPs were added first, they were able to interact immediately and emulsify the hexadecane, producing small droplets, most likely during the first emulsification using the microfluidizer. The addition of TCNF on the second emulsification, which was done at low shear, possibly did not cause significant breaking of the existing droplets and just resulted in the TCNF to being dispersed at the continuous phase, causing a large increase in viscosity. When TCNF was added first, the TCNF surrounded the hexadecane droplets (Fig. 3g), but because of its poorer wettability in hexadecane than the LNPs, larger droplets were produced. Adding LNPs to the already TCNF-covered droplets seemed not to improve the droplet size, especially with a weak shearing. The LNPs remained in the continuous phase, which was also evident by the darker color (Fig. 3a) and lower viscosity (Fig. 3d) of the TCNF-first than the LNP-first emulsion. With simultaneous mixing, both the LNPs and TCNF possibly synergistically stabilized the interface, but with the LNPs dominating the interface because of their amphiphilicity and much higher mobility than the TCNF (Fig. 3g). Despite being added simultaneously, LNPs were able to interact with hexadecane immediately and effectively because it was not bound to TCNF, which is negatively charged. This enabled the production of small droplets less susceptible to creaming. From these results, it can be deduced that the droplet size, viscosity, and stability of the emulsions can be tuned by varying the order of stabilizer addition. It should be noted, however, that the extent of shearing after the addition of the second stabilizer could yield results different from our findings.

### The effect of cationizing the LNPs

**Fig. 4:**
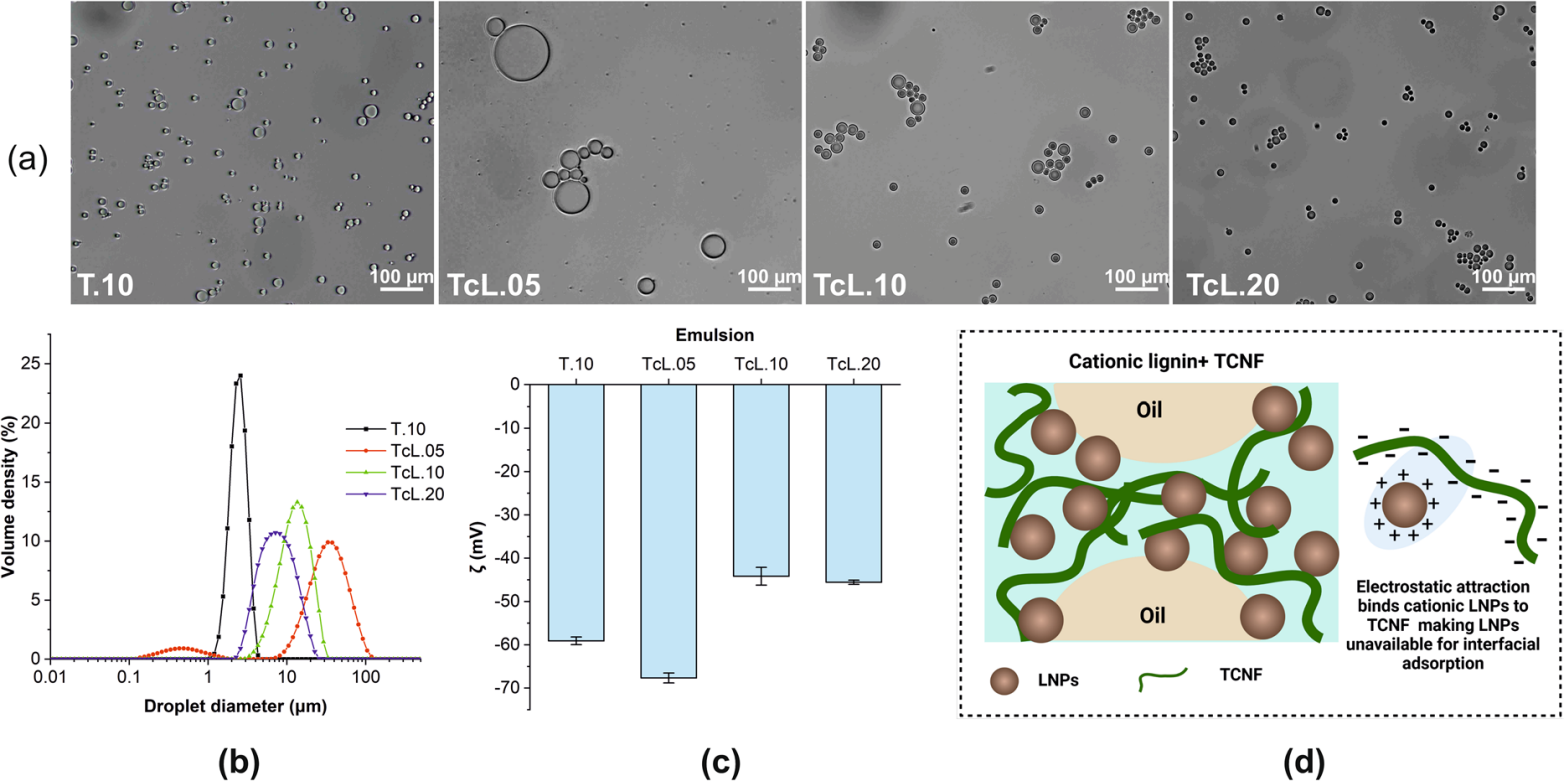
The properties of the Pickering emulsions stabilized by only 0.10% TCNF (T.10) and in combination with increasing concentration of cationic LNPs (cLNPs) at 0.05, 0.10, and 0.20% for TcL.05, TcL.10, TcL.20, respectively: appearance and droplet morphology (**a**), droplet size distribution (**b**), and zeta potential, ζ (**c**) and infographic of the interface (**d**)

The effect of electrostatically binding the LNPs by using cLNPs to form electrostatic attractions to the carboxyl groups of the TCNF was investigated. The cLNPs were produced by coating the LNP surface with lignin cationized by GTAC (Agustin et al. 2022). Our hypothesis is that the attachment of cLNPs provides permanent hydrophobic groups to TCNF, similar in effect to chemical hydrophobization. Contrary to our expectations, adding cLNPs to TCNF emulsion resulted in the formation of large, flocculated droplets, which showed a decreasing size with an increasing weight percentage of cLNPs (Fig. 4a). The individual droplets of TcL.20 were smaller than that in T.10 as seen in the optical images, but this was not reflected in the light scattering results (Fig. 4b). This is because, with light scattering, the agglomerated droplets could be detected as one large droplet, causing a discrepancy. The ζ of all the emulsions was still negative despite the addition of cLNPs, indicating that the amount of added cLNPs was not sufficient to completely neutralize the charge of the TCNF (Fig. 4c). However, with increasing cLNP content, it was evident that the ζ became less negative but were all still below − 30 mV. Thus, the evident flocculation and large droplets, especially for TcL.05 and TcL0.10, could not be explained by the change in ζ but likely due to the decrease in the availability and mobility of the spherical cLNPs to interact at the oil interface because they were electrostatically bonded to TCNF (Fig. 4d). The unbound part of the spherical cLNPs, may have also caused electrostatic attractive forces between droplets, resulting in flocculation. It is also possible that during microfluidization, the high-pressure shearing caused random release of the bound cLNPs, which then act as a linking agent between negatively charged droplets. These findings further supported our inference that the formation of small droplets was dependent on the interaction of the LNPs with hexadecane rather than that of the TCNF.

### Pickering emulsion-templated foams and use as adsorbents

One of the potential uses of PEs is that they can be templated to produce macroporous monolithic foams (Capron and Cathala 2013; Pascaud et al. 2022; Zhu et al. 2020). In addition, unique functionalities to the templated foams were achieved when two types of particles are used to prepare the PEs (Moreau et al. 2016; Voisin, et al. 2021). To investigate the additional benefit of incorporating LNPs into TCNF-based emulsions, PE-templated foams with only TCNF (TL0) or in combination with LNPs (TL.8) were prepared and used as adsorbents for pharmaceuticals, which are emerging pollutants of high concern in the aquatic environment. Nanocelluloses and their chemically-modified derivatives have shown promising potential as adsorbents for various types of water pollutants (Köse et al. 2020; Wang 2018) but the combination of TCNF and LNPs as bio-based adsorbents is relatively unexplored.

**Fig. 5:**
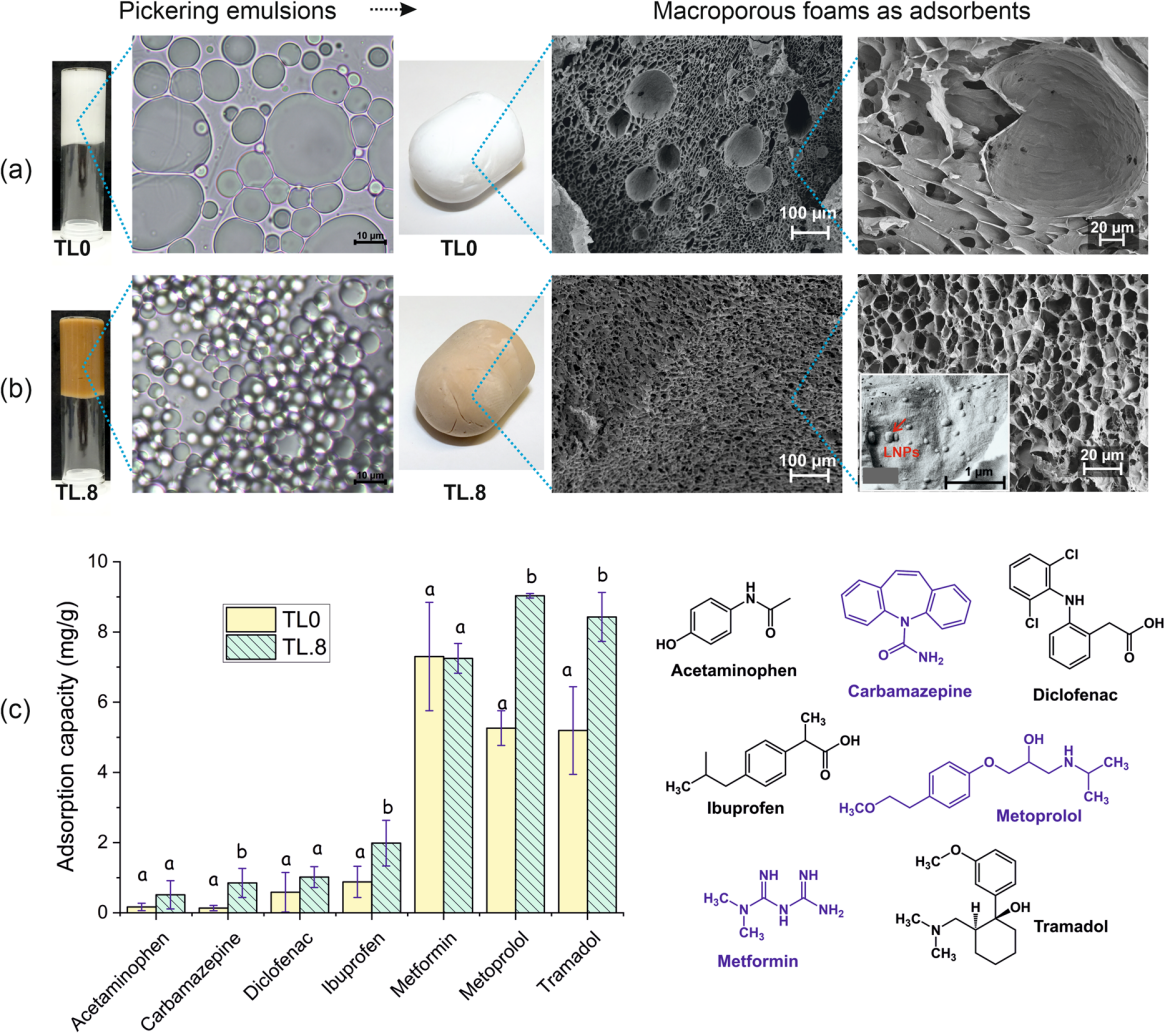
The appearance and droplet morphology of the Pickering emulsions stabilized by only TCNF (TL0, **a**), and by LNPs and TCNF (TL.8, **b**) and the subsequent foams, with respective FESEM images, produced from them, and the performance of the foams as adsorbents for different pharmaceuticals (**c**). Different letters above each bar in (c) represent a significant difference in adsorption capacity between TL0 and TL.8 for each pharmaceutical at 0.05 significance level

The production of PE-templated foams relies on the formation of PEs that can be dried without a significant collapse in the droplet structure. This was achieved both from the PEs stabilized by only 0.4% TCNF (TL0) or in combination with LNPs (TL.8) at an LNP:TCNF ratio of 0.8:1 and with 30% cyclohexane as the oil phase (Figs. 5a and 5b). As mentioned previously, the ratio is based on our previous findings with cLNPs and TCNF cryogels. Both emulsions were highly viscous and did not easily flow in inverted vials. As expected, the emulsion with LNPs (TL.8) had much smaller droplets (D_*(4,3)*_ = 28 µm) than that without LNPs (TL0), which had D_*(4,3)*_ of 60 µm. With freeze drying, lightweight porous foams were produced without visible shrinkage relative to the emulsion volume, even though the initial emulsion did not show highly connected droplets typical for Pickering high-internal phase emulsions (HIPEs). PE-templated monolithic foams were typically produced from Pickering HIPEs, which have an internal phase of more than 74%, and where the droplet interface is strengthened by polymerization or crosslinking to avoid collapse during drying (Jiang et al. 2020; Zhu, et al. 2020). However, with these PE-templated foams, the presence of unbound nanocellulose in the continuous phase provided a percolated network that support the droplet structure even after the removal of the liquid phases. The morphology (Fig. 5a and b) of the foams was affected by the emulsion droplet size. Smaller and more homogeneous pore cells were observed in TL.8 than in TL0, attributed to the effective stabilization of the emulsion with the addition of LNPs.

A small-scale screening of the adsorption potential of the foams was performed in a multi-component system, wherein six pharmaceuticals, each at 20 mg/L concentration, were dissolved in Milli-Q water. The six pharmaceuticals included acetaminophen, carbamazepine, diclofenac, ibuprofen, metformin, and metoprolol. These pharmaceuticals were selected based on their prevalence in municipal wastewater effluents in the Baltic Sea region and their differences in chemical properties and structure (Table S1) (UNESCO and HELCOM 2017). Figure 5c shows the adsorption capacity of the foams, which, in this case, is the amount of pharmaceuticals in milligrams adsorbed per gram of the foam. Because all the components, i.e., the TCNF and LNPs, in the foam were negatively charged at pH 6 due to deprotonated carboxyls, both foams exhibited a preference towards the cationic metoprolol and metformin. The removal rate for these cationic pharmaceuticals ranged from 40 to 60% while for those being neutral and aromatic (acetaminophen and carbamazepine) or anionic and aromatic (diclofenac and ibuprofen), the removal rates were less than 15%. Both foams had a similar capacity to adsorb the aliphatic cationic metformin, but they differed significantly in their performance toward the aromatic cationic pharmaceutical metoprolol. The LNP-containing foam TL.8 had a much higher adsorption capacity towards metoprolol than that of TL0. This indicates that apart from electrostatic attraction, the addition of LNPs promoted the removal of cationic aromatic compounds by other interactions such as π − π aromatic ring or hydrophobic interactions. When metoprolol was replaced with tramadol, also an aromatic cationic pharmaceutical, the results again showed a significantly higher adsorption capacity of TL.8. This is consistent with our previous study that LNPs, because of their polyaromatic structure, adsorbs cationic aromatic pharmaceuticals at a higher level than the aliphatic cationic ones (Agustin et al. 2022). Despite its negative surface, TL.8 foam also showed improved adsorption towards the aromatic and neutral carbamazepine and towards the aromatic and anionic ibuprofen, which affirmed possible removal mechanisms other than electrostatic interactions. However, there was no significant improvement for the neutral and highly polar acetaminophen and negatively charged and aromatic diclofenac. Nevertheless, the addition of LNPs to the nanocellulose-based PE-templated foams clearly added functionalities, making the material a multimodal adsorbent for improved removal of selected pharmaceuticals.

## Conclusion

Utilizing bio-based Pickering stabilizers from sustainable and renewable raw materials contributes to the UN Sustainable Development Goal towards green manufacturing and processes. It would be even better if the bio-based stabilizers could be used readily, without laborious and expensive modification procedures to provide the functionalities needed for a particular application. In this study, the high hydrophilicity of TCNF was overcome by adding naturally amphiphilic LNPs as co-stabilizers in oil-in-water PEs.

The findings revealed that the formation of stable PEs characterized by small highly negatively charged droplets that were less susceptible to creaming was highly dependent on the amount and availability of the LNPs to interact with the oil phase. The higher the amount of the LNPs, the smaller the droplets and the more stable the emulsion was. The effective pH range was 5 to 8, where the LNPs were not agglomerated and were sufficiently mobile to stabilize the droplet interface. At pH 3, the LNPs agglomerated due to protonation of the carboxyl groups, resulting in large flocculated droplets. Adding the LNPs before the TCNF or simultaneously mixing them allowed the LNPs to interact immediately with the oil phase producing stable PEs with differing viscosity. Approaches that restrict the LNPs to be adsorbed at the interface such as adding the TCNF before the LNPs or by electrostatically binding the cationized LNPs to the TCNF resulted in less stable PEs.

The LNPs acted as modifying agents to the subsequent TCNF-based foams produced by freeze drying the emulsion. The macroporous foams have potential as adsorbents for pharmaceutical pollutants and the addition of LNPs promoted the removal of aromatic pharmaceuticals of varying ionic character.

Overall, this work demonstrated the potential of LNPs as co-stabilizers of nanocellulose-based PEs, at the same time, as modifying agents to the subsequent foams. Utilizing LNPs provides a means to customize the properties and stability of the PEs without additional chemical derivatization to modify the surface properties of the nanocellulose. This approach clearly opens a green and sustainable way of stabilizing biphasic systems and obtaining materials that can be useful in many applications including those related to food, medical or environmental industries.

### Supplementary Information

Below is the link to the electronic supplementary material.

Supplementary file1 (DOCX 1001 KB)

## Data Availability

Data available from the authors upon request.
