# Supplementary material for: Lignin nanoparticles as co-stabilizers and modifiers of nanocellulose-based Pickering emulsions and foams
Source: Cellulose (Lond). 2023 Jul 29;30(14):8955–71. doi: 10.1007/s10570-023-05399-y (PMC10509128; doi:10.1007/s10570-023-05399-y)
Supplement: Supplementary file 1 — Supplementary file1 (DOCX 1001 KB) [file 10570_2023_5399_MOESM1_ESM.docx]

Lignin nanoparticles as co-stabilizers and modifiers of nanocellulose-based Pickering emulsions and foams

Melissa B. Agustin,*^a^ Neda Nematollahi,^a^ Mamata Bhattarai,^a,b^ Erfan Oliaei,^c^ Mari Lehtonen,^a^ Orlando J. Rojas,^b,d^ and Kirsi S. Mikkonen^a,e^

^a^ Department of Food and Nutrition, Faculty of Agriculture and Forestry, University of Helsinki P.O. Box 66, FI-00014, Helsinki, Finland

^b^ Department of Bioproducts and Biosystems, Aalto University, P.O. Box 16300, 00076, Aalto, Finland

^c^ Wallenberg Wood Science Center, Department of Fiber and Polymer Technology, KTH Royal Institute of Technology, SE-100 44 Stockholm, Sweden

^d^ Bioproducts Institute, Department of Chemical and Biological Engineering, Department of Chemistry and Department of Wood Science, University of British Columbia, 2360, East Mall, Vancouver, BC V6T 1Z3, Canada

^e^ Helsinki Institute of Sustainability Science, University of Helsinki, P.O. Box 65, FI-00014, Helsinki, Finland

* Corresponding author e-mail: melissa.agustin@vtt.fi

Present affiliation: VTT Technical Research Centre of Finland Ltd., P.O. Box 1000, FIN-02044 VTT, Finland

**SUPPORTING INFORMATION**

**Fig. S1.** Number of published documents related to the keyword “Pickering emulsions” per year retrieved from Scopus Database in February 2023.


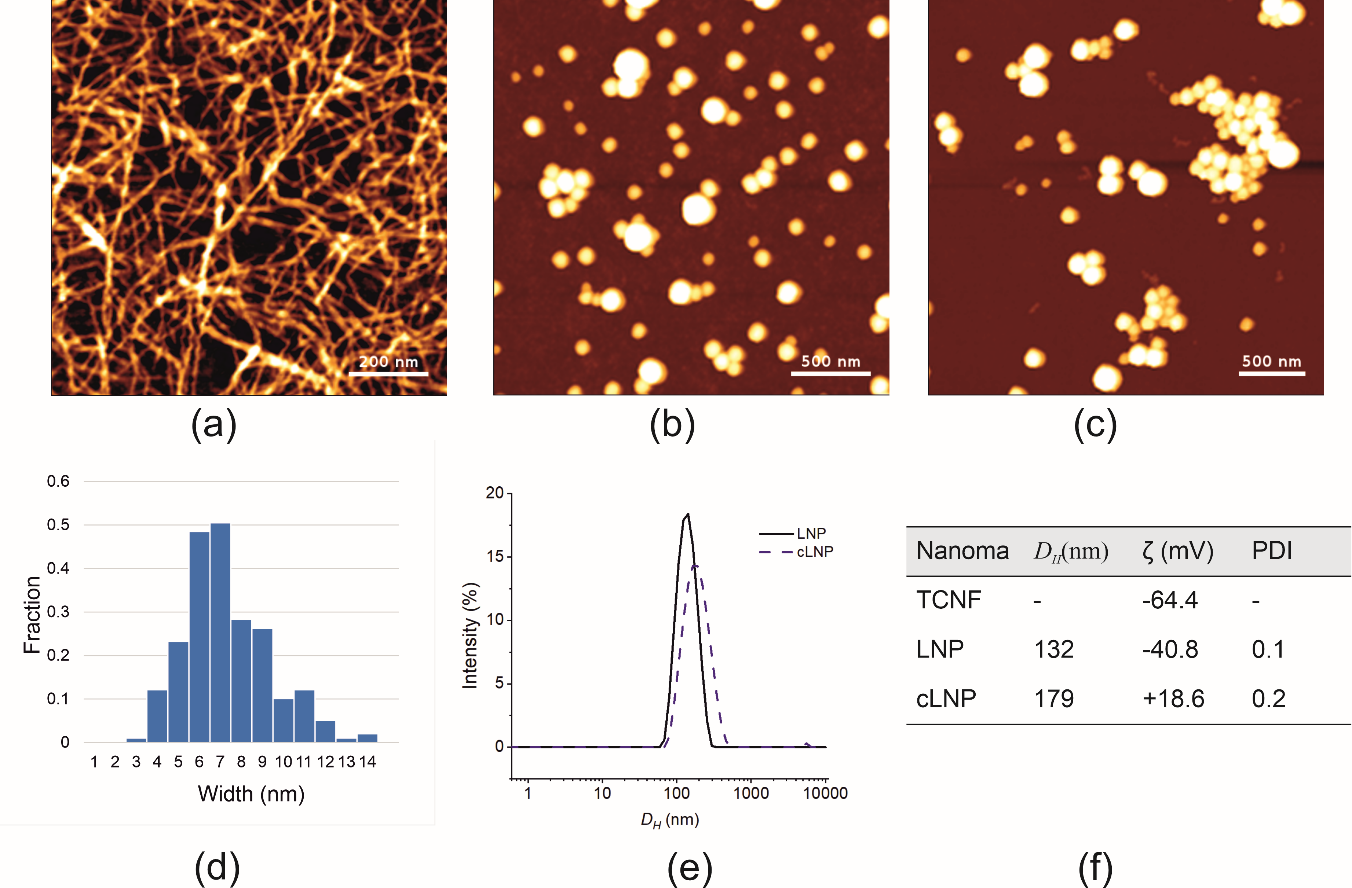


**Fig. S2.** AFM images of TCNF (a), LNPs (b), cLNPs (c), and width and hydrodynamic diameter distribution (*D_H_*) of TCNF (d), LNPs and cLNPs (e) and average *D_H_*, zeta potential, ζ, and polydispersity indexes, PDI, (f) of the different nanomaterials.


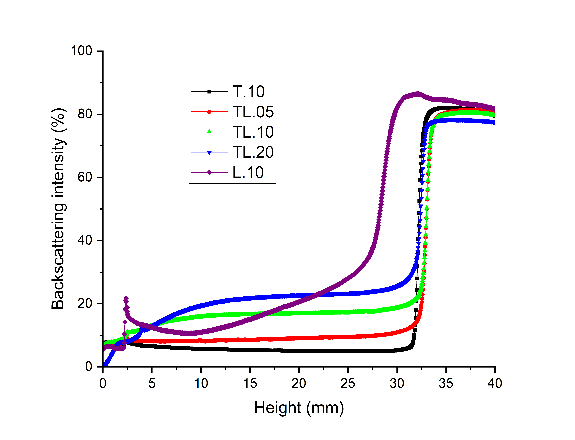

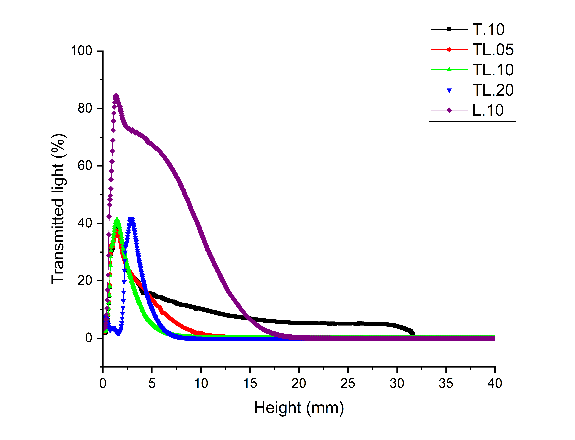


(a) (b)

**Fig. S3.** Backscattering and transmission profiles of Pickering emulsions stabilized by only 0.1% TCNF (T.10), only 0.1% LNP (L.10), and their combinations with increasing LNP content (TL.05, TL.10, TL.20).

**Table S1.** The list of pharmaceuticals used in the adsorption experiment and their properties

| Active pharmaceutical ingredient, abbreviation and structure | CAS  number | Form in water | pKa* | log K_ow_^*^ |
| --- | --- | --- | --- | --- |
| Acetaminophen, ACE   | 103-90-2 | Neutral | 9.4 | 0.46 |
| Carbamazepine, CBZ   | 298-46-4 | Neutral | 14 | 2.5 |
| Diclofenac, DCF   | 15307-86-5 | Anionic | 4.2 | 4.5 |
| Ibuprofen. IBU   | 15687-27-1 | Anionic | 4.9 | 4.0 |
| Metformin, MTF   | 657-24-9 | Cationic | 12 | -2.6 |
| Metoprolol, MPL   | 37350-58-6 | Cationic | 9.6 | 1.9 |
| Tramadol, TRA   | 27203-92-5 | Cationic | 9.4 | 3.0 |

Acid form pKa and logK_ow_ values were obtained from a database available at https://pubchem.ncbi.nlm.nih.gov/

**S1. Preparation and characterization of TCNF, LNPs and cationic LNPs**

**Preparation of TCNF.** The preparation followed the established method of TCNF preparation described by Saito, et al. (Saito, et al. 2007) with slight modfication. The never-dried pulp fibers were beaten with a PFI mill for 2000 revolutions according to ISO 5264-2:2011. The beaten fibers were dipped into an HCl solution (pH 2) for 30 min and then washed with deionized water and filtered off several times until pH neutralized. Then, a 1% water suspension of wood fibers was prepared and pH was adjusted to 10 using 1 M NaOH and 0.5 M HCl solutions. Afterward, a pre-dissolved solution containing 6 mmol/g pulp of NaBr and 80 µmol/g pulp of TEMPO was added to pulp suspension while mechanical stirring. Then, 6 mmol NaClO/g pulp was added dropwise to the suspension, pH adjusted to 10.5 and the reaction was continued for 60 min. The pulp was then washed with deionized water, filtered until the washing reached a neutral pH. After which, a 1% pulp suspension was homogenized with a high-pressure microfluidizer (Microfluidizer M-110EH, Microfluidics Corp., USA). The first homogenization pass was with two serial Z-shaped interaction chambers with path diameters of 400 and 200 µm at a pressure of about 600 bar, followed by 2 passes at interaction chambers with path diameters of 200 and 100 µm at a pressure of about 1600 bar. The solid content of the produced TCNF suspension was 0.7%.

**Preparation of LNPs.** The anti-solvent nanoprecipitation method using acetone was used to prepare the LNPs (Figueiredo, et al. 2021). A three-gram lignin was dissolved in 300 mL of 3:1 (v/v) acetone/water and stirred at 500 rpm at room temperature for 2 h. The lignin solution was filtered using a glass microfibre filter (Whatman GF/F, pore size 0.7 ‎µm‎) to remove undissolved solids, and the filtrate was then poured into 500 mL of MilliQ-water which was magnetically stirred at 600 rpm. The mixture was evaporated under reduced pressure at 40 °C to remove the acetone. The LNP dispersion was further stirred overnight in an open vessel to further concentrate the suspension, which later gave a lignin content of about 5.6 mg/mL.

**Preparation of cationic lignin nanoparticles (cLNPs).** Soluble cationic lignin was first prepared by dissolving in a round-bottom flask 5 g lignin in 50 mL 0.2 M NaOH. The solution was stirred at 70 rpm at 70 ‎‎℃ and GTAC (5.5 g) was added dropwise. The reaction was maintained for 1 h, after which, the pH was adjusted to 7 by 0.2M sulfuric acid (Kong, et al. 2015). The resulting mixture was then dialysed (Spectra/Por 1, 1 kDa MWCO) against deionized water for 3 days, changing the water periodically. The dialysed cationic lignin was mixed with anionic LNPs at a dry weight ratio of 0.3:1 cationic lignin to LNPs to produce the cLNPs. Finally, the cLNP dispersion was dialysed using Spectra/Por 7 (6-8 kDa MWCO) against deionized water for the duration of four days to remove any residual non-adsorbed cationic lignin (Agustin, et al. 2022; Sipponen, et al. 2017).

**Characterization of the nanomaterials.** The zeta potential (ζ) of the LNPs, cLNPs, and TCNF were measured at 25 ℃ with a Zetasizer Nano ZS (Malvern Instruments, Worcestershire, UK). The diluted samples were loaded in folded capillary cells. The hydrodynamic diameter, D*_H_*, was analysed using the same instrument. All experiments were performed with 3 samples. Intensity-based average D*_H_* and ζ values were used in reporting the data*.* Atomic force microscopy (AFM) imaging was performed to visualize the morphology of the nanomaterials using the Multimode AFM Nanoscope 9 (Bruker, Germany). The PPP-FMaud-10 by Nanosensors with a spring constant of 0.5 – 9.5 N/m was used to probe the samples using the quantitative imaging mode program. The width of the TCNF was measured using scanning probe microscopy software Gwyddion.

**REFERENCES**

Agustin MB, Mikkonen KS, Kemell M, Lahtinen P, Lehtonen M (2022) Systematic investigation of the adsorption potential of lignin- and cellulose-based nanomaterials towards pharmaceuticals. Environmental Science: Nano 9:2006-2019

Figueiredo P, Lahtinen MH, Agustin MB, de Carvalho DM, Hirvonen SP, Penttila PA, Mikkonen KS (2021) Green Fabrication Approaches of Lignin Nanoparticles from Different Technical Lignins: A Comparison Study. ChemSusChem 14:4718-4730

Kong F, Parhiala K, Wang S, Fatehi P (2015) Preparation of cationic softwood kraft lignin and its application in dye removal. European Polymer Journal 67:335-345

Saito T, Kimura S, Nishiyama Y, Isogai A (2007) Cellulose Nanofibers Prepared by TEMPO-Mediated Oxidation of Native Cellulose. Biomacromolecules 8:2485-2491

Sipponen MH, Smyth M, Leskinen T, Johansson L-S, Österberg M (2017) All-lignin approach to prepare cationic colloidal lignin particles: stabilization of durable Pickering emulsions. Green Chemistry 19:5831-5840
